# Three-Dimensional Rotation, Twist and Torsion Analyses Using Real-Time 3D Speckle Tracking Imaging: Feasibility, Reproducibility, and Normal Ranges in Pediatric Population

**DOI:** 10.1371/journal.pone.0158679

**Published:** 2016-07-18

**Authors:** Li Zhang, Jing Zhang, Wei Han, Jun Gao, Lin He, Yali Yang, Ping Yin, Mingxing Xie, Shuping Ge

**Affiliations:** 1 Department of Ultrasound, Union Hospital, Tongji Medical College, Huazhong University of Science and Technology, Wuhan, China; 2 Department of Ultrasound, Wuhan Women and Children Medical Center, Wuhan, China; 3 Epidemiology and Health Statistics, Huazhong University of Science and Technology, Wuhan, China; 4 The Heart Center, St. Christopher's Hospital for Children/Drexel University College of Medicine, Philadelphia, PA, United States of America; 5 Deborah Heart and Lung Center, Browns Mills, NJ 08015, United States of America; University of Cincinnati, College of Medicine, UNITED STATES

## Abstract

**Background and Objective:**

The specific aim of this study was to evaluate the feasibility, reproducibility and maturational changes of LV rotation, twist and torsion variables by real-time 3D speckle-tracking echocardiography (RT3DSTE) in children.

**Methods:**

A prospective study was conducted in 347 consecutive healthy subjects (181 males/156 females, mean age 7.12 ± 5.3 years, and range from birth to 18-years) using RT 3D echocardiography (3DE). The LV rotation, twist and torsion measurements were made off-line using TomTec software. Manual landmark selection and endocardial border editing were performed in 3 planes (apical “2”-, “4”-, and “3”- chamber views) and semi-automated tracking yielded LV rotation, twist and torsion measurements. LV rotation, twist and torsion analysis by RT 3DSTE were feasible in 307 out of 347 subjects (88.5%).

**Results:**

There was no correlation between rotation or twist and age, height, weight, BSA or heart rate, respectively. However, there was statistically significant, but very modest correlation between LV torsion and age (R^2^ = 0.036, *P*< 0.001). The normal ranges were defined for rotation and twist in this cohort, and for torsion for each age group. The intra-observer and inter-observer variabilities for apical and basal rotation, twist and torsion ranged from 7.3% ± 3.8% to 12.3% ± 8.8% and from 8.8% ± 4.6% to 15.7% ± 10.1%, respectively.

**Conclusions:**

We conclude that analysis of LV rotation, twist and torsion by this new RT3D STE is feasible and reproducible in pediatric population. There is no maturational change in rotation and twist, but torsion decreases with age in this cohort. Further refinement is warranted to validate the utility of this new methodology in more sensitive and quantitative evaluation of congenital and acquired heart diseases in children.

## Introduction

Left ventricular (LV) rotation, twist, and torsion, due to the complex helical myocardial fiber architecture, are the result of the clockwise rotation of the base and the counterclockwise rotation of the apex of the LV and play an important role in ventricular systolic and diastolic performance [1–6]. Only recently has it become possible to measure left ventricular rotation, twist and torsion in the clinical setting using echocardiography and cardiac magnetic resonance (CMR) [7–9]. Two-dimensional speckle-tracking echocardiography (2D STE) has proven to be a simple non-invasive technique to quantify the LV rotation, twist, and torsion, and has been validated by CMR tagging and sonomicrometry in the adult [8,9] and pediatric ​[10–15] population. However, 2D STE is limited by a number of intrinsic limitations, including loss of speckles due to motion outside the imaging plane and suboptimal reproducibility.

More recently, three-dimensional speckle-tracking echocardiography (3D STE) has been developed to overcome the limitations of 2D STE [12–17]. This method tracks the motion of speckles within the scan volume, allowing more complete and accurate assessment of myocardial deformation in all three spatial dimensions by avoiding the loss of speckles due to out-of-plane motion. However, there are trade-offs of 3D STE, such as compromises of spatial and temporal resolution of 3DE and 3D STE.

The specific aims of our study were: 1) to evaluate the feasibility and reproducibility of LV 3D rotation, twist and torsion by RT 3D STE, and 2) to establish the maturational changes and normal values of these measures in a large prospective pediatric cohort.

## Methods

### Study Population

The study was approved by the institutional research ethics committee at Union Hospital, Tongji Medical College, Huazhong University of Science and Technology, China. All data used were anonymized. The subjects gave written informed consent. All procedures and data analysis were performed by the authors; specific contributions of all enlisted authors are provided.

A total of 347 consecutive subjects from birth to 18 years were prospectively enrolled at Union Hospital in Wuhan, China. The inclusion criteria included: 1) referral for echocardiographic evaluation for clinical reasons, 2) no previous history of congenital or acquired heart disease, and 3) completely normal studies on 2D and Doppler echocardiography with normal structure, chamber size, wall thickness and systolic function. Exclusion criteria included: 1) structural congenital or acquired heart disease, 2) abnormal cardiac rhythm, and 3) hypertension and/or other acute or chronic illnesses. Demographic variables, including age and gender, were collected at the time the echocardiographic studies were performed. Informed consent was obtained. Weight, height, heart rate, and blood pressure measurements were also obtained in all subjects.

### Echocardiographic Image Acquisition

All subjects had a complete transthoracic echocardiogram to determine cardiac structure, chamber size, wall thickness and cardiac function according to the recommendations of the American Society of Echocardiography. ​[18–20] All studies were performed at rest without sedation. A commercially available system (iE33, Philips Medical Systems, Andover, MA) was used with S5, S8, or S10 broadband phased-array transducers, depending on the age of the subject. Subsequently, all subjects underwent 3D echocardiographic study using the same system with either X3-1, X7-2, or X5-1 transducer. Images were optimized to obtain the entire left ventricle in a full-volume data set in the apical four-chamber view. Data sets were acquired using a four heartbeat or seven hearts beat acquisition setting. End-expiratory breath holding was performed when feasible. A minimum of four data sets were acquired for each subject, and three best quality data sets were selected for offline analysis. Data sets that missed a portion of the left ventricle, had indistinct endocardial borders, or had significant stitch artifacts were excluded.

### Echocardiographic Parameters

Cardiac chamber size, mass, and systolic LV function were measured in accordance with the recommendations for chamber quantification of the American Society of Echocardiography. [18–20] LV end-diastolic length (LVEDL), LV end-systolic length (LVESL), and mid-right ventricular diameter (RVD2) were measured in the apical 4-chamber view at the level of the left ventricular papillary muscles. The thickness of the LV septal wall and posterior wall and the LV internal dimensions were measured from the parasternal long-axis window. QLAB 3D (Philips Medical Systems) quantification software algorithms were used for semi-automated analysis of LV end-diastolic volume (LVEDV), LV end-systolic volume (LVESV), LV end-diastolic mass, LV end-systolic mass, and LV ejection fraction (LVEF), as previously described. ​[19–21]

### 3D STE

**Fig 1:**
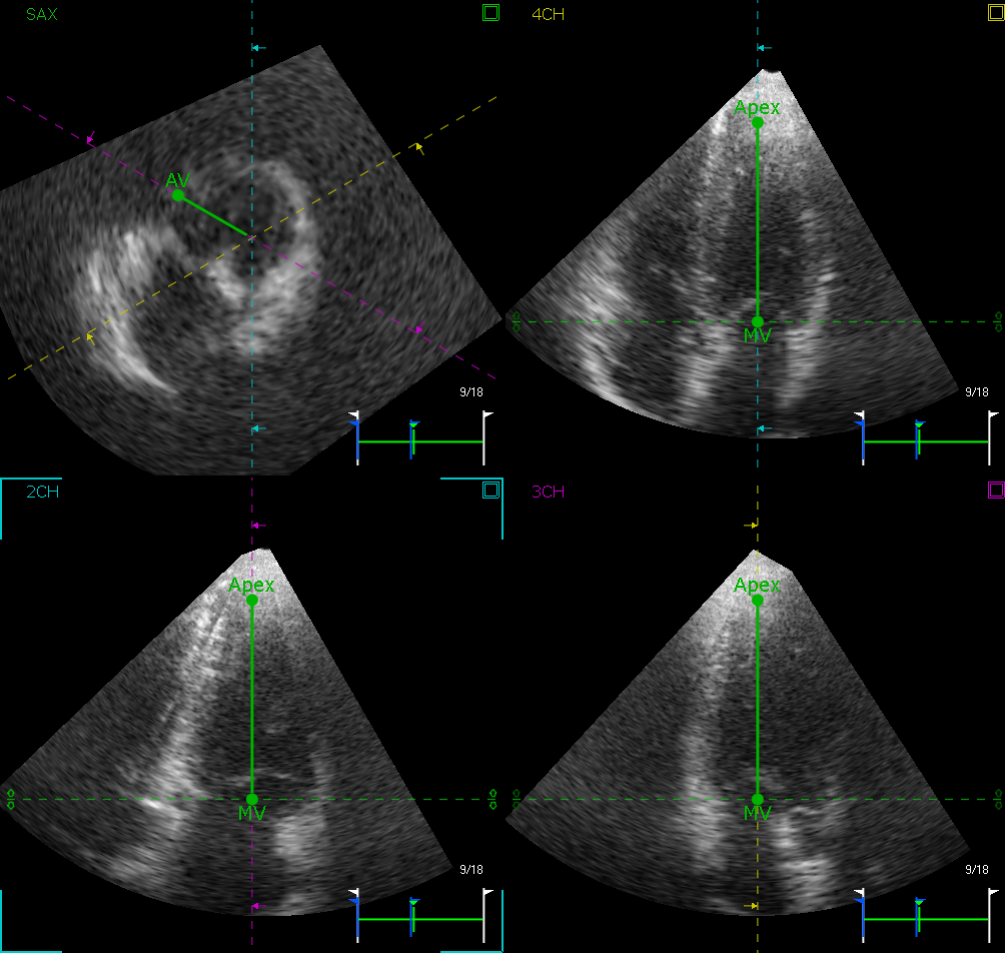
Three-dimensional speckle-tracking echocardiography (3D STE) offline analysis. First, the long axis (LAX) view is selected. The mitral valve landmark is placed at the annular level in the middle of the mitral valve, and the apical landmark is placed at the apex. Then, the short-axis view is selected. The aortic valve landmark is placed at valve level in the middle of the aortic valve. Manual tracking revision is performed if needed.

**Fig 2:**
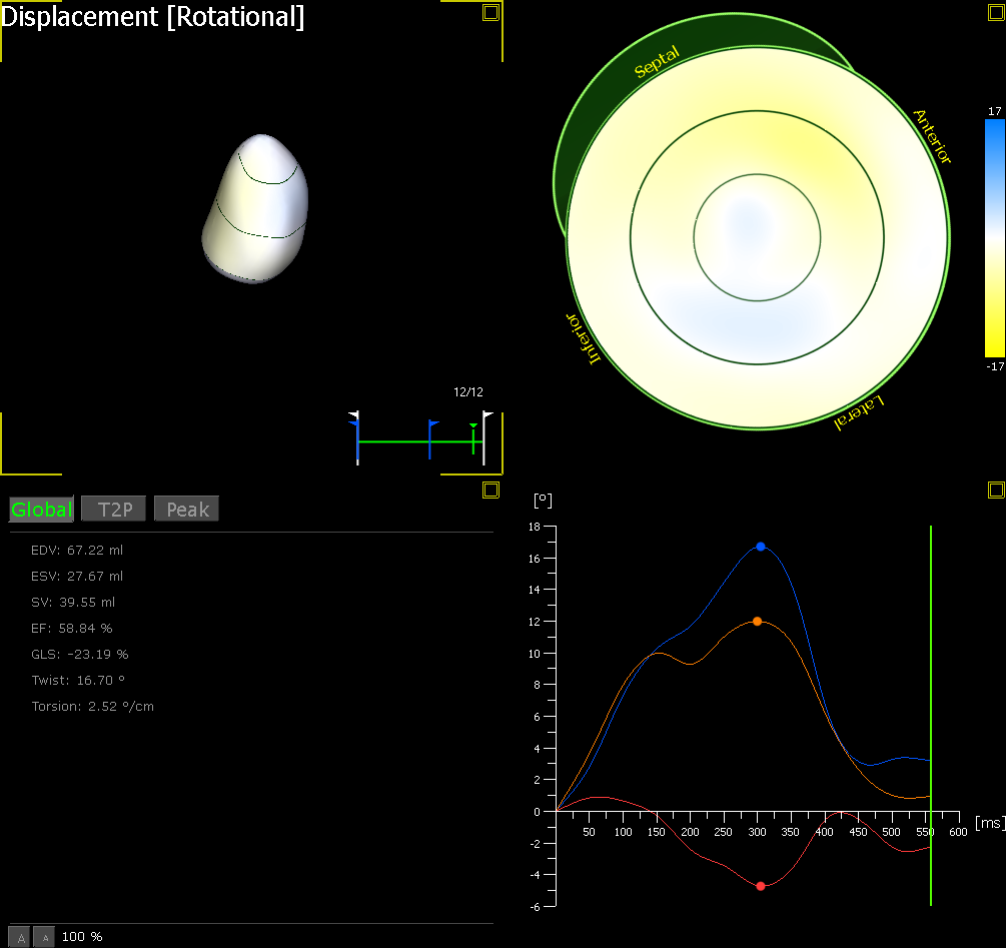
The analysis of LV rotation at apical (orange line), rotation at basal (red line) and twist (blue line) variables can be derived and displayed.

Offline 3D STE analysis was performed using software for echocardiographic quantification (4D LV-analysis 3.0; TomTec Imaging Systems, Unterschleissheim, Germany). Measurements were made using the dataset with the best image quality. The frame rate of the volumetric images was 15 to 24 frames / second. The 3D data sets were displayed as multi-planar reconstruction images corresponding to four tiles containing three standard long axis (LAX) views (apical four chamber, apical three chamber, and apical two chamber) and a short axis view, which is orthogonal to the LAX views (Fig 1). The software automatically aligned the 4D dataset such that the standard LAX views (apical four chamber, apical three chamber, and apical two chamber) were correctly displayed and the end-diastolic and end-systolic frames were automatically defined (S1 Fig). In these LAX views, the LV boundaries were first manually selected for the three anatomic landmarks (mitral annulus and LV apex). The aortic valve landmark was selected within the short-axis view in the middle of the aortic valve. Adjustments were made in the multi-planar reconstruction images until the landmarks (mitral valve, LV apex, and aortic valve) were well positioned in each standard view. The 3D endocardial surface was automatically reconstructed and tracked in 3D space throughout the cardiac cycle and manually adjusted when necessary until a best match with the actual endocardial position was visually verified in all views (S2 Fig). Subsequently, the Beutel revision state displayed a static 3D surface model of the LV (Beutel) automatically calculated by the application. Finally, the LV was automatically divided into 16 3D segments using the standard segmentation of the American Society of Echocardiography ​[18,20]. The curves and maps of the 3D LV global rotation, twist and torsion analyses were calculated (Fig 2).

By convention, counterclockwise LV rotation as viewed from the apex was expressed as a positive value, whereas a clockwise rotation was denoted as a negative value. LV twist was defined as the net difference between the basal and apical rotation angles (LV twist = apical LV rotation–basal LV rotation). LV torsion was calculated as the net LV twist normalized with respect to ventricular end diastolic (ED) longitudinal length between the LV apex and the mitral plane (LV torsion (°/cm) = LV twist /ED length). The coordinates for rotation, twist and torsion for each frame were exported to a spreadsheet (Excel 2007; Microsoft Corporation, Redmond, WA).

### Reproducibility of 3D STE Study Data

Inter-observer and intra-observer variabilities of the measurements were assessed in 50 randomly selected subjects (10 in each age group). To assess intra-observer variability, the same observer (L.Z.) measured the 3D rotation, twist and torsion analysis twice at an interval of 2 months to avoid recall bias. To assess inter-observer variability, 3D rotation, twist and torsion measurements were performed by a second observer (J.Z.), who was blinded to the results of the first observer (L.Z.).

### Statistical Analysis

All demographic, conventional echocardiographic, and 3D LV rotation, twist and torsion data are presented as numbers or percentages (mean ± SD). The relations between age and 3D LV rotation, twist and torsion variables were determined using scatter plots, one-way analysis of variance, and second-order polynomial regression analysis. Intra-observer and inter-observer agreements were calculated using the coefficient of variation (i.e., the percentage absolute difference between the measurements divided by their mean value) and intra-class correlation coefficient. *P* values < .05 were considered statistically significant. Statistical analyses were performed using SPSS for Windows version 16.0 (SPSS, Inc., Chicago, IL).

## Results

A total of 347 subjects were evaluated for inclusion in the study. Of those, LV rotation, twist and torsion analysis by RT 3D STE was feasible in 307 subjects (88.5%) (mean age, 5.9 ± 4.9 years; 161 male and 146 female subjects), whereas 40 subjects were subsequently excluded, including 15 subjects due to low frame rates as determined by the software system and 25 subjects due to the poor 3D images.

On the basis of changes of somatic growth rate in infancy (rapid growth), preschool years (deceleration in growth), preadolescence (relatively steady growth), onset of puberty (beginning of growth spurt), and adolescence (heterogeneous growth in early, middle, and late adolescence), the effects of age and cardiac growth on LV functional indices were examined. The final 307 study subjects were divided into 5 representative age groups [11]: group 1 (birth to 1 year of age), group 2 (1–5 years of age), group 3 (6–9 years of age), group 4 (10–13 years of age), and group 5 (13–18 years of age)(Table 1).

**Table 1:** Demographic and anthropometric characteristics.

	Age group				
	1 (< 1 y)	2 (≥ 1–< 5 y)	3 (≥ 5–< 9 y)	4 (≥ 9–< 13 y)	5 (≥ 13–< 18 y)
Number	48	89	65	53	52
Male, n (%)	25 (52.1)	48 (53.4)	35(53.4)	27 (50.9)	26(50)
Age, years	0.53 ± 0.22	2.8 ± 1.08*	6.43 ± 1.13*	11.67 ± 1.31*	15.03 ± 1.69*
Height, cm	67.98 ± 5.23	92.67 ± 10.89*	124.60 ± 9.67*	144.32 ± 9.14*	164.07 ± 8.91*
Weight, kg	7.36 ± 2.12	14.45 ± 3.21*	25.32 ± 6.13*	34.23 ± 7.71*	53.29 ± 10.71*
BSA, m^2^	0.34 ± 0.05	0.60 ± 0.12*	0.94 ± 0.12*	1.14 ± 0.15*	1.53 ± 0.21*
SBP, mm Hg	81.5 ± 6.6	95.8 ± 7.6*	102.1 ± 6.4*	109.1 ± 8.8*	117 ± 9.4*
DBP, mm Hg	57.2 ± 4.9	63.8 ± 6.7*	74.3 ± 8.9*	85.9 ± 8.8*	86.5 ± 8.4
HR, beats/min	117.7 ± 11.8	103 ± 12.1*	90.1 ± 12.6*	85.2 ± 13.4*	79.8 ± 15.5

BSA body surface area; DBP, diastolic blood pressure; SBP, systolic blood pressure; HR, heart rate.

*P < .05 when compared with the younger group.

With increasing age, subject characteristics and anthropometric parameters of height, weight, body surface area, systolic and diastolic blood pressure increased (*P* < .01), and the heart rate decreased significantly (Table 1).

Conventional echocardiographic parameters of the study subjects are presented in Table 2. Age differences were noted in LV structural and functional variables (*P* < .05), except for LVEF which remained unchanged throughout the age groups (*P* > .05).

**Table 2:** Conventional echocardiographic variables.

Variable	Age group					
	1 (<1 y)	2 (≥1-<5 y)	3 (≥5-<9 y)	4 (≥9-<13 y)	5 (≥13-<18 y)	*P*
LVEDL, cm	3.68 ± 0.43	4.32 ± 0.59	5.32 ± 0.57	5.79 ± 0.65	6.79 ± 1.11	< .0001
LVESL, cm	2.93 ± 0.41	3.17 ± 0.47	4.14 ± 0.46	4.54 ± 0.52	5.23 ± 0.89	< .0001
LVIDd, cm	2.24 ± 0.18	2.82 ± 0.37	3.38 ± 0.55	3.86 ± 0.44	4.12 ± 0.49	< .0001
SWT, cm	0.41 ± 0.07	0.49 ± 0.06	0.54 ± 0.08	0.68 ± 0.12	0.77 ± 0.09	< .0001
PWT, cm	0.32 ± 0.04	0.37 ± 0.05	0.46 ± 0.06	0.52 ± 0.09	0.57 ± 0.08	< .0001
RVD2, cm	1.71 ± 0.21	2.01 ± 0.28	2.42 ± 0.37	2.87 ± 0.36	2.96 ± 0.40	< .0001
LVEDV, mL	14.82 ± 5.48	24.39 ± 7.51	42.43 ± 12.45	56.34 ± 15.16	82.23 ± 27.74	< .0001
LVESV, mL	5.72 ± 2.24	8.78 ± 3.08	15.43 ± 4.76	22.24 ± 56.18	29.27 ± 15.23	< .0001
LV EF, %	62.32 ± 4.65	63.71 ± 5.55	63.32 ± 4.54	63.67 ± 5.34	64.21 ± 4.07	0.7276
ED mass, /m^2^	25.98 ± 11.34	38.09 ± 12.23	64.09 ± 19.51	78.67 ± 19.18	136.38 ± 39.39	< .0001
ES mass, g/m^2^	15.32 ± 6.79	22.38 ± 6.59	38.21 ± 10.26	48.41 ± 16.27	81.85 ± 23.17	< .0001

LV, left ventricular; LVEDL, LV end-diastolic length; LVESL, LV end-systolic length; LVID, LV internal dimension; SWT, interventricular septal wall thickness; PWT, left ventricular posterior wall thickness; RVD2, mid-right ventricular diameter; LVEDV, LV end-diastolic volume; LVESV, LV end-systolic volume; LV EF, LV ejection fraction; ED mass, mass at end diastole; ES mass, mass at end-systole.

Data are expressed as mean ± SD.

### Normal ranges and maturational changes of 3D LV rotation, twist and torsion variables

Three-dimensional LV rotation, twist and torsion data are presented in Table 3. There was no correlation between rotation or twist with age. However, there was a statistically significant, but very modest correlation between LV torsion and age (R^2^ = 0.036, *P*< .001). There was no significant difference in rotational and twist variables among the age groups but there were statistically significant differences in torsion (*P*< .001) among the 5 age groups, except for the analysis between group 5 and group 4 as well as between group 4 and group 3. Table 4 shows the normal values of the LV rotation and twist for the different age groups and Table 5 shows those of LV torsion in this cohort. There was no statistical difference between the genders for all 3D LV rotation, twist and torsion variables.

**Table 3:** Three-dimension LV rotation and torsion of study subjects.

Variable	Age group					
	Group 1 (<1 y)	Group2 (≥1-<5 y)	Group3 (≥5-<9 y)	Group4 (≥9-<13 y)	Group5 (≥13-<18 y)	*P*
Rotation-apical (°)	8.20 ± 5.22	5.63 ± 3.58	6.52 ± 3.86	5.64 ± 3.91	5.91 ± 3.41	.0726
Rotation- basal (°)	-3.68 ± 1.53	-4.50 ± 2.09	-4.19 ± 2.24	-4.31 ± 1.87	-3.91 ± 1.99	.0943
Twist (°)	11.89 ± 4.96	10.14 ± 4.14	10.71 ± 4.52	9.96 ± 4.37	9.82 ± 3.29	.2343
Torsion (°/cm)	3.08 ± 0.97*^2,3,4,5^	2.01 ± 0.89*^1,3,4,5^	1.64 ± 0.81*^1,2,5^	1.30 ± 0.87*^1,2,^	0.97 ± 0.72*^1,2,3^	.0001

*P< .05 (age groups denoted by superscript numerals).

**Table 4:** Normal ranges of LV twist and rotation of study subjects.

Variable	N	Mean ± SD	95% percentile
Rotation-apical (°)	307	6.26±5.05	(1.49,13,35)
Rotation- basal (°)	307	-4.65±2.21	(-7.89, -1.54)
Twist (°)	307	10.75±4.18	(4.69,19.08)

**Table 5:** Normal ranges of LV torsion of study subjects by age group.

Age Group	N	Mean ± SD	95% percentile
1(<1 y)	48	3.08±0.97	(4.87, 1.09)
2(≥1-<5 y)	89	2.01±0.89	(3.81, 0.21)
3(≥5-<9 y)	65	1.64±0.81	(3.31, -0.02)
4(≥9-<13 y)	53	1.30±0.87	(2.69, -0.12)
5(≥13-<18 y)	52	0.97±0.72	(1.73, 0.06)

### Intra-observer and inter-observer variability

Intra-observer and inter-observer variability data are shown in Tables 6 and 7. The intra-observer and inter-observer variabilities for apical and basal rotation, twist and torsion ranged from 7.3% ± 3.8% to 12.3% ± 8.8% and from 8.8% ± 4.6% to 15.7% ± 10.1%, respectively. Interclass correlation coefficients ranged from 0.78 to 0.89 and from 0.76 to 0.83 for intra-observer and inter-observer measurements for rotation, twist and torsion, respectively.

**Table 6:** Intra-observer variability of LV twist, torsion and rotation by 3DE.

	Observer 1	Observer 1	CV (%)	ICC
Rotation-apical (°)	5.81±3.88	5.37±4.19	12.3±8.8	0.83
Rotation- basal (°)	-4.38±3.84	-4.91±3.32	8.5±7.9	0.89
Twist (°)	1.95±0.98	1.97±0.10	7.3±3.8	0.81
Torsion (°/cm)	12.01±5.02	13.23±5.78	9.1±4.5	0.78

**Table 7:** Inter-observer variability of LV twist, torsion and rotation by 3DE .

	Observer 1	Observer 2	CV (%)	ICC
Rotation-apical (°)	5.56±3.98	6.17±3.75	15.7±10.1	0.79
Rotation- basal (°)	-5.17±3.65	-4.78±4.43	9.4±8.3	0.83
Twist (°/cm)	1.91±0.87	2.08±0.10	8.8±4.6	0.78
Torsion	12.76±6.23	11.67±7.08	11.7±6.3	0.76

## Discussion

To the best of our knowledge, this is the largest prospective pediatric study to investigate the feasibility and reproducibility of 3D STE and a modality independent software and algorithm to derive LV 3D rotation and twist variables. We also investigated normal values and maturational changes in this large pediatric cohort.

### Normal range and maturational change in LV rotation, twist and torsion

Previous studies have reported an increase in LV apical, basal rotation, and twist​[12] and a decreasing trend in LV torsion ​[13] from infants to adults. These studies had small sample sizes (n = 45 and 142, respectively) and even smaller subgroups in young infants and adolescences (n = 25 from 9 days to 16 years and n = 2 of the 5 groups from 3 to 16 years, respectively). Both studies used 2D STE and custom-designed algorisms and software to calculate rotation, twist and torsion. Our study demonstrated that in spite of the slight heterogeneity of the LV rotation and twist from neonates to adolescents, the maturational changes were not statistically significant in this large prospective cohort using 3D STE. Therefore, it is plausible that the maturational decreasing torsion is due to the growth of the longitudinal dimension of the LV as torsion is the normalized twist by the LV length from apex to the base. This is consistent with our perspective study that demonstrated there were statistically significant but clinical irrelevant maturational changes in 3D global, longitudinal, radial and circumferential strain in the normal pediatric population​[14]. These data suggest that although maturational growth in LV chamber size continues through the growth spurt and adolescence, LV functional maturation, e.g. EF, strain, rotation, twist and torsion, occurs fairly early and as early as newborns. The mechanisms underlying these findings may be explained by well accepted notion that the number of ventricular myocytes is established at birth although more recent data suggest that myocytes undergo continuous turnover and dying cells are constantly replaced by newly formed myocytes​[22].

### 2D STI vs 3D STI

Echocardiographic speckle tracking is a technique based on a frame-by-frame tracking of speckle patterns created by interference of the ultrasound beam within the myocardial tissue​[4,5]. 2D STE is based on standard B-mode imaging and is therefore not dependent on the angle or translation of the ventricle with excellent temporal resolution. 2D STE has been well validated for LV rotation, twist and torsion measurements by sonomicrometry and tagged magnetic resonance imaging​[8–10]. However, limitations of the 2D strain technique have been well recognized. First, two-dimensional speckle tracking analysis cannot track the three dimensional motion of the heart, thus, cannot eliminate the errors introduced by through-plane motion. Second, the exact location of the basal and apical plane may vary from patient to patient, which affects the reproducibility of 2D STE.

### Feasibility and reproducibility of 3D STE

Our results showed measurement of LV rotation, twist and torsion is highly feasible by the 3D STE technique (88.5%). The intra-observer and inter-observer variabilities for apical and basal rotation, twist and torsion ranged from 7.3% to 15.7%. Previous studies suggested that the good reproducibility is due to the fact that 3D STE is not limited to one imaging plane and, as a result, can better track the complex 3D motion of the LV speckles​[5,8,9,17].

### Potential clinical applications

Although the clinical utility of rotation, twist and torsion is still unfolding, there are growing evidence and investigations using this new approach to assess LV function in various cardiovascular disorders in both adults and children​[23–25]. More recently, it has been shown that E/E’, TDI E velocity, and torsion abnormalities existed in the early postoperative stage of transposition of the great arteries after arterial switch operation​[26]. Acute afterload reduction in patients undergoing balloon angioplasty for coarctation of the aorta or congenital valvar aortic stenosis resulted in significant improvement in torsion, preceding other changes in radial and circumferential deformation ​[27]. Van at al ​[28] showed that in hypertrophic cardiomyopathy, there is a different pattern of rotation at the base vs the apex, depending on whether there was sigmoid septum or a reversed septal curvature pattern.

In addition, there are many other studies in the literature that showed that LV mechanical parameters are altered in congenital and acquired heart diseases. For example, Menting E et al found that the majority of patients with corrected tetralogy of Fallot showed a reduced LV twist mainly due to decreased apical rotation​[29]. Strikingly, one-quarter of these patients have an abnormal apical rotation which is associated with decreased systolic LV and RV function. Dong et al also found that LV systolic twist significantly improved after transcatheter secundum atrial septal defect (ASD) closure​[30]. This improvement was mainly attributed to the improved LV basal rotation. In a third study, Tejman-Yarden et al found that in structurally normal hearts, significant losses in the basal rotation, LV twist and longitudinal strain as assessed by speckle tracking echocardiography, occur during acute AV synchronous RV pacing​[31]. Although these are studies in smaller populations and using other speckle tracking techniques, they showed that the LV mechanical parameters are altered in many congenital and acquired heart defects with potential clinical applicability.

### Limitations

There are a few limitations related to this study. First, the left ventricular rotation, twist, and torsion by3D STE technology has not been validated against other reference standard. Second, the ethnic group we studied is limited to the Asian population. It has been noted in the published literature that suggested there is variability in LV volume, mass, and function among certain ethnic groups​[32]. Further research is required to evaluate the variability of 3D STE for assessment of LV rotation, twist and torsion variables in children of various ethnic backgrounds. Third, an important limitation of the current 3D STE in the determination of torsional mechanics is the inability to determine the rates of twisting and untwisting. The inability to determine these parameters limits the usefulness of this technique to assessing diastolic recoil and function and possible maturational changes with continuous switching of titin isoforms from fetal life to adulthood. Finally, 3D STE technology will likely evolve and improve in temporal resolution, sector size and width, and more modality independent and automated 3D STE computational algorithms. Future advances may further facilitate the clinical use of this technology as a promising sensitive and robust tool for the assessment of various congenital and acquired heart diseases before and after medical, percutaneous, or surgical interventions.

## Conclusion

The measurement of LV rotation, twist and torsion by this new RT 3D STE methodology is feasible and reproducible in children. There is no maturational change in rotation and twist, but torsion decreases with age in this cohort. Further refinement is warranted to validate the utility of this new methodology in congenital and acquired heart diseases.

## Supporting Information

S1 Fig The software automatically aligned the 4D dataset such that the standard LAX views (apical four chamber, apical three chamber, and apical two chamber) were correctly displayed and the end-diastolic and end-systolic frames were automatically defined. (TIF)

S2 Fig Tracking revision workflow. (TIF)

## Abbreviations

2D: Two-dimensiona
3D: Three-dimensiona
CI: Confidence interva
EDL: End-diastolic length
EDV: End-diastolic volume
EF: Ejection fraction
ESL: End-systolic length
ESV: End-systolic volume
LAX: Long-axis
LVEF: left ventricular ejection fraction
RT: Real-time
RVD: Right ventricular diameter
STE: Speckle tracking echocardiography
