# Supplementary figures and images for: Three-Dimensional Rotation, Twist and Torsion Analyses Using Real-Time 3D Speckle Tracking Imaging: Feasibility, Reproducibility, and Normal Ranges in Pediatric Population
Source: PLoS One. 2016 Jul 18;11(7):e0158679. doi: 10.1371/journal.pone.0158679 (PMC4948847; doi:10.1371/journal.pone.0158679)

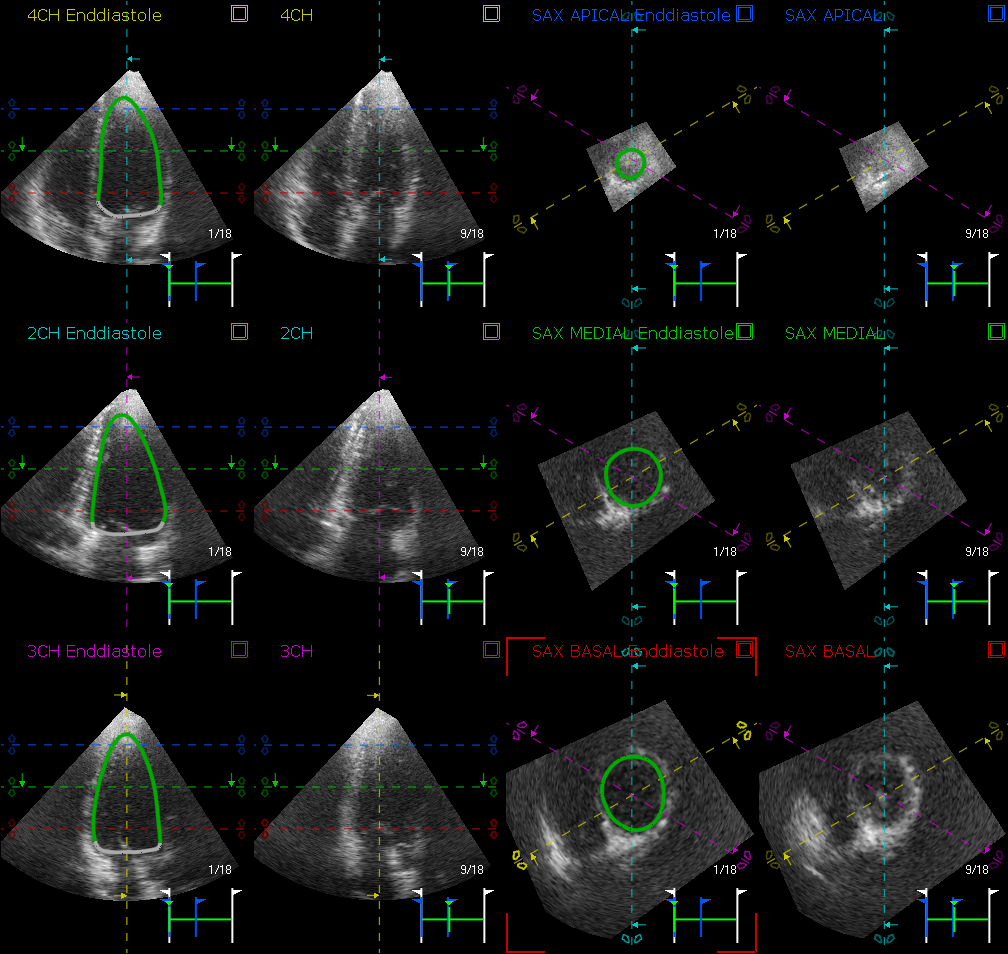

Supplement: S1 Fig — (TIF) [file pone.0158679.s001.tif]

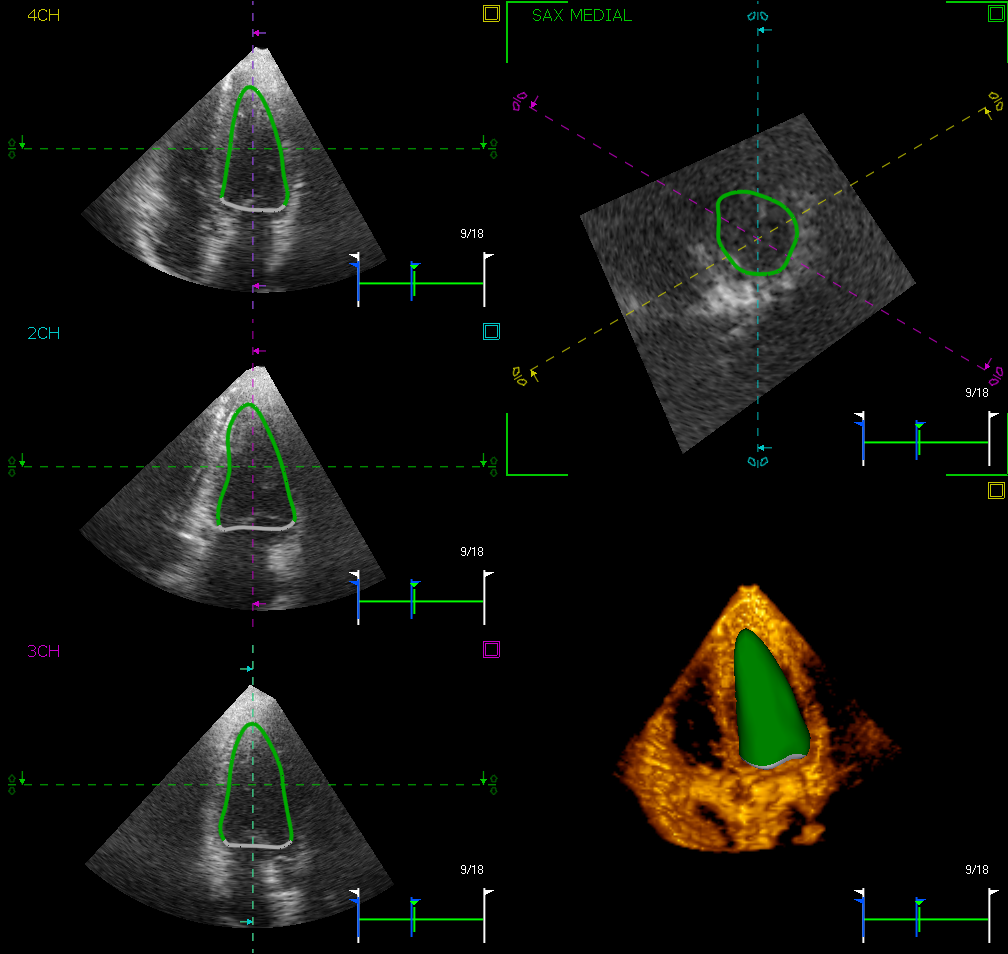

Supplement: S2 Fig — (TIF) [file pone.0158679.s002.tif]
